# An artificial self-assembling peptide with carboxylesterase activity and substrate specificity restricted to short-chain acid p-nitrophenyl esters

**DOI:** 10.3389/fchem.2022.996641

**Published:** 2022-09-19

**Authors:** Yanfei Liu, Lili Gan, Peili Feng, Lei Huang, Luoying Chen, Shuhua Li, Hui Chen

**Affiliations:** ^1^ Key Laboratory of Cell Engineering of Guizhou Province, Affiliated Hospital of Zunyi Medical University, Zunyi, China; ^2^ The Clinical Stem Cell Research Institute, Affiliated Hospital of Zunyi Medical University, Zunyi, China; ^3^ Collaborative Innovation Center of Chinese Ministry of Education, Zunyi Medical University, Zunyi, China

**Keywords:** self-assembling peptide, artificial enzyme, esterase-like, nanofiber, substrate specificity, competitive inhibitor

## Abstract

Natural enzymes possess remarkable catalytic activity and high substrate specificity. Many efforts have been dedicated to construct artificial enzymes with high catalytic activity. However, how to mimic the exquisite substrate specificity of a natural enzyme remains challenging because of the complexity of the enzyme structure. Here, we report artificial carboxylesterases that are specific for short chain fatty acids and were constructed via peptide self-assembly. These artificial systems have esterase-like activity rather than lipase-like activity towards p-nitrophenyl esters. The designer peptides self-assembled into nanofibers with strong β-sheet character. The extending histidine units and the hydrophobic edge of the fibrillar structure collectively form the active center of the artificial esterase. These artificial esterases show substrate specificity for short-chain acids esters. Moreover, 1-isopropoxy-4-nitrobenzene could function as a competitive inhibitor of hydrolysis of p-nitrophenyl acetate for an artificial esterase.

## Introduction

Enzymes are highly evolved macromolecular biocatalysts with remarkable catalytic activity and substrate specificity. They are highly desired in chemical, medical, biological, and industrial fields. The amino acid residues of an enzyme govern its three-dimensional structure. This structure in turn controls substrate-specific binding and catalysis (Wang et al., 2018). *De novo* design of enzymes remains difficult of the complexity of the primary sequence folding process (Woolfson et al., 2015; Huang J. et al., 2016). In the past decades, many efforts have been made toward artificial enzymes (Liu et al., 2020; Han et al., 2021). While enzyme-like activity has been described via successful construction of catalytically active sites, very few studies (Qu et al., 2017; Makam et al., 2019; Zhu et al., 2019; Li et al., 2020) have attempted to mimic the substrate specificity of an enzyme due to the complexity of the recognition center.

Self-assembling peptides can undergo spontaneous organization into well-ordered amphiphilic nanostructures driven by noncovalent interactions, and have been widely used in three-dimensional cell culture, sustained release, tissue engineering and nanotechnology (Wychowaniec et al., 2020a; Zhang, 2020). The common features of the peptide assemblies share some of the characteristics of enzymes and suggest that these peptides are a promising framework to mimic natural enzymes and provide a backbone of active sites (Zozulia et al., 2018; Liu et al., 2020; Han et al., 2021). Compared to the natural enzymes, these peptide assemblies are much more robust and stable over a broad range of pH and temperature and can be readily synthesized (Singh et al., 2021; Dowari et al., 2022). Histidine has been frequently found in active center of proteases and other hydrolytic enzymes as its imidazole side chain is capable of serving as a hydrogen bond donor or acceptor (Sharma et al., 2016). Previously, various catalytic sequences such as histidine, His-Ser-Gly, and Ser-His-Asp were incorporated into a supramolecular framework to mimic the catalytic function of native esterase (Guler and Stupp, 2007; Wang G. et al., 2016; Zhang et al., 2016; Garcia et al., 2017; Zhang et al., 2017; Huang et al., 2020; Gazit et al., 2021; Dowari et al., 2022). Impressively, a series of histidine-containing amyloid-forming peptides were capable of catalyzing ester hydrolysis with remarkable efficiency in the absence of Zn^2+^ (Rufo et al., 2014; Song et al., 2018; Díaz-Caballero et al., 2020). However, to the best of our knowledge, very few self-assembling peptide-based esterases offer a substrate selectivity similar to natural esterases (Makam et al., 2019).

**SCHEME 1:**
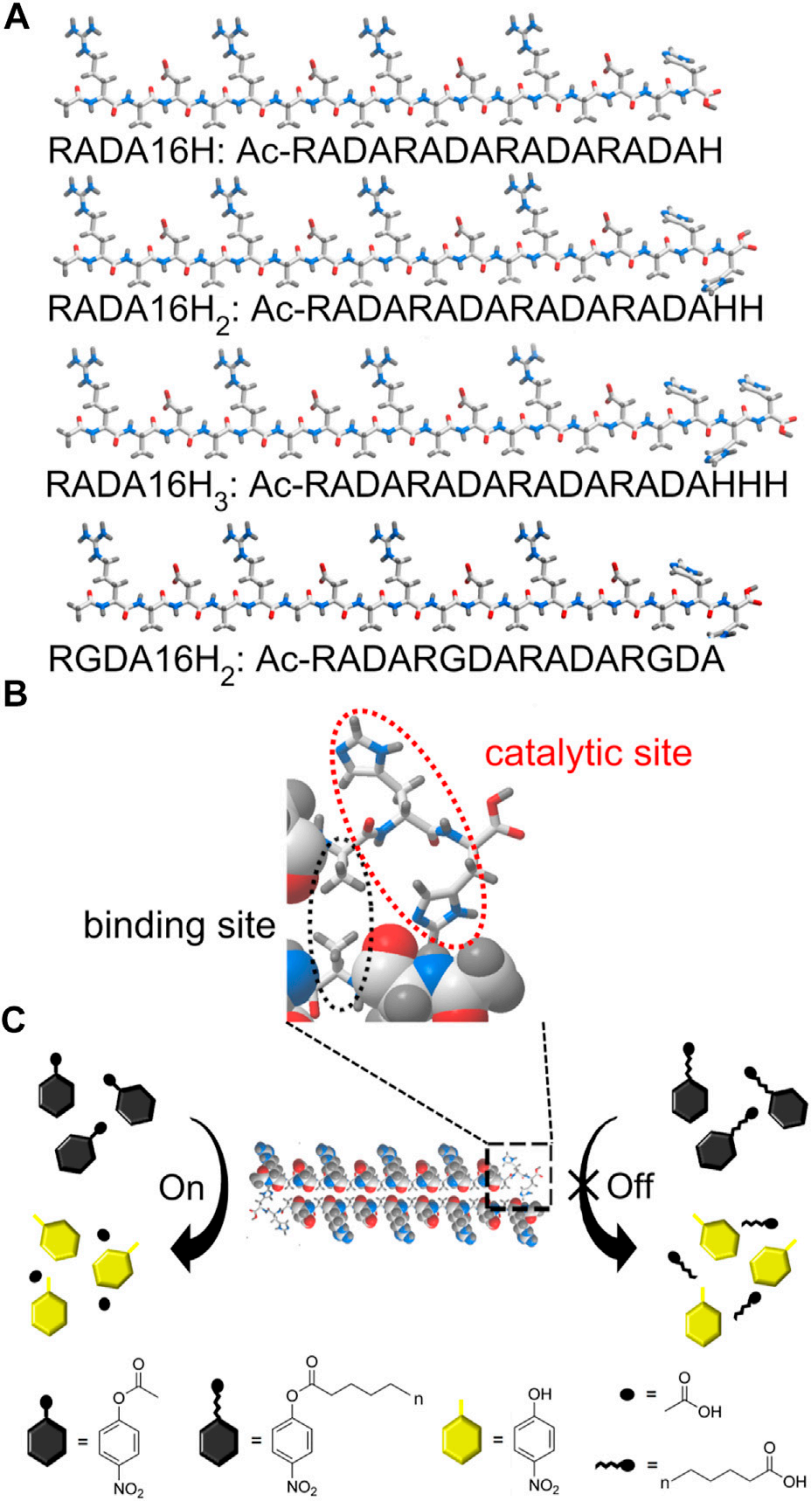
**(A)** Schematic of the designer peptide. **(B)** An enlarged view of the indicated area of detail containing the active center of the artificial esterase based on peptide RADA16H_2_. The edge of the self-assembled β-sheet bilayer functions as a binding site for short-chain esters through hydrophobic interactions. The two histidines are located on the edge of the nanofiber with a high degree of internal order to catalyze the hydrolysis of the substrates. **(C)** Substrate specificity of the nanofibers formed by peptide RADA16H_2_ (shown as cross section) towards p-nitrophenyl esters. RADA16H_2_ shows preferential hydrolytic activity towards short-chain acids esters containing 2 to 4 carbon atoms.

Here we report an example of histidine-bearing self-assembling peptides as an artificial carboxylesterase (EC 3.1.1.1). These systems are specific for short chain fatty acids (<10 carbons). These peptides showed esterase-like activity rather than lipase-like activity towards p-nitrophenyl esters (Chang et al., 2021). A series of histidine-functionalized RADA16 peptides were obtained by incorporating histidine residues into the supramolecular framework to catalyze the hydrolysis of p-nitrophenyl esters (Scheme 1A). These designer peptides were named RADA16H, RADA16H_2_, and RADA16H_3_. RADA16 is an classic self-complementary peptide (RADARADARADARADA-CONH_2_) that can undergo spontaneous assembly into well-ordered nanofibers by hydrophobic collapse of the hydrophobic surface of its β-sheet (Zhang et al., 1994; Zhang 2020). The β-sheet structure formed by RADA16 was quite stable over a wide pH range (Ye et al., 2008). The hydrophobic surface of RADA16 can encapsulate and release various hydrophobic molecules based on the hydrophobic interaction in aqueous solution (Briuglia et al., 2014; Lu and Unsworth, 2016), and the hydrophobic residues on the exposed edge of the self-assembled fibrillar structure (Bowerman et al., 2011; Wychowaniec et al., 2020b) provides a substrate-binding site through non-specific hydrophobic interactions (Zhang et al., 2017). Histidines were chosen as the catalytic sites and extended from the nanofibers into the solution (Scheme 1B). RGDA16 (RADARGDARADARGDA-CONH_2_) in which two alanines were substituted for glycines was reported to adopt a random coil conformation and hampered the self-assembling of the peptide and formation of the β-sheet (Zhang et al., 2005). Thus two histidines were covalently connected to the C-termini of RGDA16 to create a control peptide RGDA16H_2,_ which is expected to have a weak affinity to the hydrophobic substrate (Scheme 1B).

We first confirmed the importance of the secondary structure of the peptide to the esterase mimics. These peptides showed an esterase-like activity (Scheme 1C) that catalyzes the hydrolysis of short-chain acyl p-nitrophenyl esters such as pNPA and pNPB. Furthermore, compounds similar to pNPA were used to study the hydrophobic interactions between the substrate and the artificial esterase. Finally, the inhibition study showed that 1-isopropoxy-4-nitrobenzene (INB) could be a competitive inhibitor of the artificial esterase.

## Materials and methods

### Materials

pNP phosphate and all the p-nitrophenyl esters were purchased from Sigma-Aldrich. 4-methylimidazole was purchased from Acros Organics. 1-Isopropoxy-4-nitrobenzene and p-nitrophenyl glycerol were obtained from TCI America and Alfa aesar, respectively. The peptides RADA16H, RADA16H_2_, RADA16H_3_ and RGDA16H_2_ were commercially synthesized form SciLight Biotechnology LLC and used without further purification.

### Preparation of nanofibers

Peptide stock solutions were prepared by dissolving the peptide powers with Mill-Q water (18.2 MΩ) at a concentration of 5 mM. All the peptide solutions were sonicated for 15 min and then kept in room temperature for 24 h to ensure nanofiber network formation.

### Transmission electron microscopy

The peptide samples were diluted to 0.5 mM and incubated overnight at room temperature. Before observation, 10 μl of peptide solution was dropped on the surface of a copper grid covered by a perforated formvar film and negatively stained with stained with uranyl acetate. After drying, the grid was observed directly under transmission electron microscope (Hitachi-7650).

### Atomic force microscopy

RADA16H_2_ stock solution was diluted with 50 mM buffer solutions of pH (acetate for pH 5.0, borate for pH 9.0 and 10.1) to 0.5 mM for AFM imaging. After incubation overnight at room temperature, 5 μl of peptide RADA16H_2_ sample was deposited on silicone substrates for 30 s. Then the samples were washed with Mill-Q water and air-dried. The images were obtained using AFM (Bruker Dimension ICON) in tapping mode.

### CD analysis

The peptide solutions were diluted to a concentration of 0.075 mM and CD spectrum between 190 and 260 nm was recorded on a Model 400 CD Spectrophotometer (Aviv Biomedical, Inc.) at 25°C. All spectra were corrected by subtracting the baseline, and the data were expressed as mean residue ellipticity, [θ], which was given in the units of degrees per cm^2^/dmoL. The CD spectra of the peptides were analyzed by the SELCON3 program from the CDPro package (Colorado State University) to analyze the secondary structure contents of the peptides, using the IBasis7 [SDP48] reference proteins set.

### Fluorescence spectroscopy

The measurements of ThT fluorescence were performed using a Fluoromax-4 fluorescence spectrophotometer (Horiba Scientific) with excitation of 450 nm. For each measurement, the freshly prepared peptide solutions were diluted in PBS buffer (pH7.4) to 1.65 mM and incubated at RT overnight and then mixed with ThT solutions. The final concentration of ThT was 5 μM.

### Rheology properties

Rheology experiments of the gels were performed at 25°C on a rheometer (HAAKE Rheostress I) with a cone and plate geometry system (cone diameter 2 cm, angle 1°, truncation 51 μm, gap 25 mm). An aliquot of 100 μl of 5 mM of peptide solution was placed on the plate. Then 100 μl of PBS (pH7.4) buffer was dropped around the peptide aliquot. After gelation for 20 min, the PBS solution was removed and time sweeps were performed at constant shear stress of 1 Pa and constant frequency of 6 rad/s for 20 min. Temperature control was provided with a temperature regulated circulating water bath (Haake Phoenix II).

### Preparation of hydrogel and hydrogel catalytic reaction

100 μl of 5 mM RADA16H solution was pipette into the center of a well from a 24-well plate. 250 μl 50 mM PBS (pH 7.4) buffer was gently added on the top of the scaffold to induce gelation. The system was incubated at 25°C for 30 min. Then the buffer inside the well was removed and hydrogel was washed by PBS buffer (pH 7.4). 3 × 10^-6^ moL of pNPA was dissolved in 500 μl of PBS buffer, added on top of the hydrogel and left to diffuse and react at 25°C for 6 h. Then the concentration of p-nitrophenol was analyzed by HPLC (Shimadzu LC20A) using InerSustain C18 column with a SPD-M20A diode array detector at 244 nm. Because pNPA was poorly soluble in water, solutions of substrate were dissolved in acetonitrile. The concentration of acetonitrile in all reaction mixture was 1%.

### Kinetics measurements

Hydrolysis kinetics were monitored at 400 nm and 25°C by adding the appropriate amount of p-nitrophenyl acetate or the other p-nitrophenyl esters in acetonitrile to the buffer solution containing the catalysts. Before each measurement, peptide stock solutions of RADA16H, RADA16H_2_, RADA16H_3_ and RGDA16H_2_ were freshly diluted to a concentration of 10^−4^ M. In a typical experiment, the final concentration of substrate varied from 2 × 10^−4^ M to 6.4 × 10^−3^ M. As for competitive inhibition assay, the final concentration of 1-Isopropoxy-4-nitrobenzene varied from 2 × 10^−4^ M to 10 × 10^−3^ M. Because of the poor solubility in aqueous solutions, the p-nitrophenyl esters and pNPA resembling compounds were dissolved in acetonitrile before added to the buffer solutions. The concentration of acetonitrile in all reaction mixture was 1%.

UV-Vis spectra (Evolution 300) data were converted to product concentration by Beer-Lambert law. The observed hydrolysis rate (V_obs_) was corrected for the buffer rate by subtracting the rate obtained in buffer solution with no catalyst present (V_uncat_). The extinction coefficients for the hydrolysis product p-nitrophenol at different pH values were experimentally obtained by measuring the absorbance of p-nitrophenol in the various buffers. Kinetic parameters were obtained from Michaelis–Menten plots of V_net_/[S] (V_net_ = V_obs_-V_uncat_) measured with catalytic nanofibers (V_obs_) or buffer alone (V_uncat_). The Linweaver-Burke plots of 1/V_net_ against 1/[S] were used as further verification. The first-order rate constants of hydrolysis k_cat_ is given by k_cat_ = V_max_/[E], where [E] is the concentration of peptides. The second-order rate constants K_2_ were calculated from liner regression of the measured pseudo first-order rate constants as a function of 4-methylimidazole or peptide RGDA16H_2_. As for the inhibition assay, Dixon analysis was used to determine the inhibitory type and the inhibitory constant (K_i_). All the catalytic experiments were repeated three times and the data were calculated as means ± SD.

## Results and discussion

### Characterization of peptide assemblies

**FIGURE 1:**
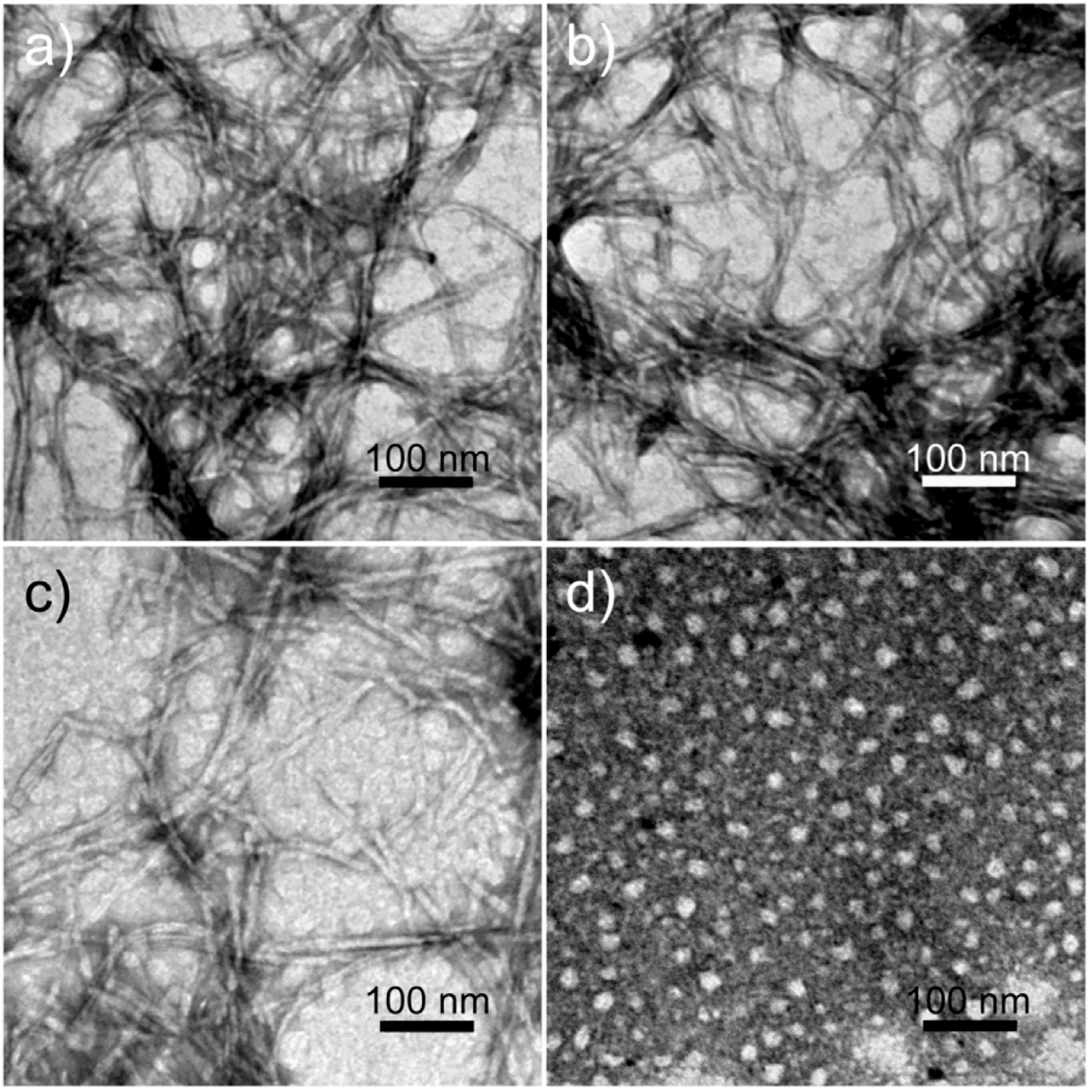
TEM images of nanostructures formed from 0.1 mM **(A)** RADA16H, **(B)** RADA16H_2_, **(C)** RADA16H_3_, and **(D)** RGDA16H_2_. The bar represents 100 nm.

**FIGURE 2:**
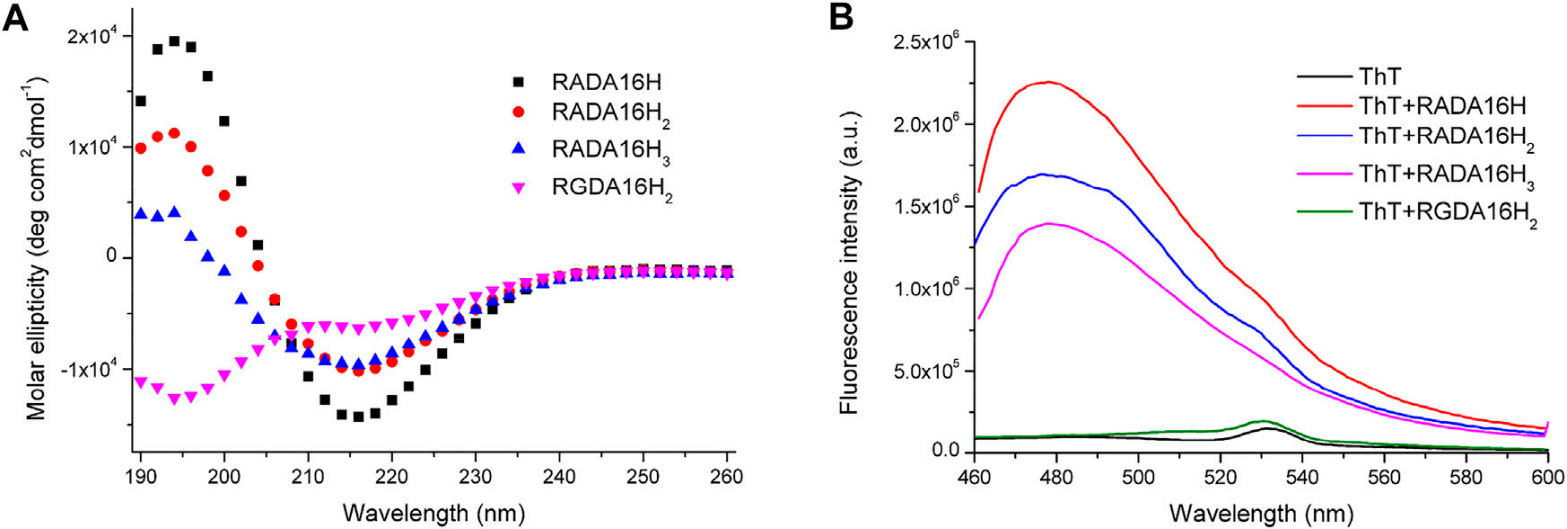
**(A)** CD spectrum of 75 μM RADA16H, RADA16H_2_, RADA16H_3_, and RGDA16H_2_ dissolved in water. CD signals were collected at 25°C. Peptides RADA16H, RADA16H_2_, and RADA16H_3_ form β-sheet structures while RGDA16H_2_ is a random coil structure; **(B)** The fluorescence emission of 5 μM ThT in pH 7.4 buffer solution with 1.65 mM RADA16H, RADA16H_2_, RADA16H_3_, and RGDA16H_2_.

All peptides were soluble in pure water. The peptides RADA16H, RADA16H_2_, and RADA16H_3_ self-assembled into high aspect ratio nanofibers with widths of ∼7 nm and lengths from hundreds of nanometers to several micrometers (Figures 1A–C). The observed width is consistent with estimates of the width of the β-sheet formed by the collapsed hydrophobic alanine group. Concurrently, most RGDA16H_2_ formed polydisperse nanospherical aggregates with diameters of 10–30 nm (Figure 1D). Figure 2A shows the circular dichroism spectra of RADA16H, RADA16H_2_, and RADA16H_3_; the results demonstrated a sharp minimum at 216 nm indicating the predominance of β-sheets. According to the CDpro analysis, the β-sheet contents of RADA16H, RADA16H_2_, RADA16H_3_ and RGDA16H_2_ were 60.1%, 42.6%, 34.7% and 9.3% (Supplementary Table S1), respectively. The β-sheet content is negatively related to the number of the attached histidines. Meanwhile, RGDA16H_2_ adopts an irregular secondary structure. This observation was confirmed by a thioflavin T (ThT) binding assay to study β-sheet surface formation (Figure 2B). ThT did not produce the characteristic fluorescent signal when incubated with RGDA16H_2_, but there was signal at 480 nm with nanofibers formed from RADA16H, RADA16H_2_, or RADA16H_3_ (Bagrov et al., 2016; Chen et al., 2018). The affinities of ThT for these nanofibers are positively related to the β-sheet content of the peptides.

These data clearly indicate that the predominant presence of β-sheets was crucial for peptide self-assembly and nanofiber formation. In accordance with these results, 5 mM of peptides RADA16H, RADA16H_2_, and RADA16H_3_ formed hydrogels due to fibrillar entanglement and crosslinking after exposure to equal volumes of PBS (pH 7.4). The mechanical strength of the hydrogels decreased significantly as more histidine residues were extended to the C-termini of the peptide RADA16 (Supplementary Figure S1) (Wychowaniec et al., 2020a).

### Hydrolysis kinetic study of the peptide nanofibers

Next, p-nitrophenyl acetate (pNPA) was used as the substrate for esterase activity because it is a common model compound to study the catalytic activity of natural enzymes and artificial catalysts. Catalytic tests were initially performed by adding 3 × 10^−5^ mol of pNPA directly on top of the 100 μl of 5 mM RADA16H hydrogel at 25°C. After 6 h of incubation, the reactions were decanted and directly analyzed by HPLC. We found that >96% of the substrate was converted to the p-nitrophenol product (Supplementary Figure S2).

**FIGURE 3:**
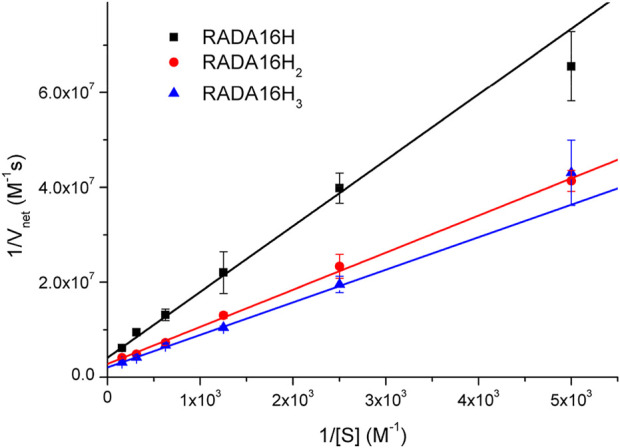
Lineweaver-Burke plots of the hydrolysis of various concentrations of pNPA catalyzed by 0.1 mM of RADA16H, RADA16H_2_ and RADA16H_3_ at 25°C.

To further understand the catalytic mechanism of the artificial esterase, we examined the dependence of the initial rate on the substrate concentration while keeping the concentration of enzymes at 0.1 mM. Hydrolysis of pNPA was monitored by UV-vis spectroscopy at 400 nm to follow the formation of p-nitrophenol. Under our conditions, the hydrolysis rates of pNPA catalyzed by RADA16H, RADA16H_2_, and RADA16H_3_ at pH 7.4 were measured by varying the concentration of pNPA. The double-reciprocal plots of the initial rate versus pNPA concentration are linear (Figure 3), indicating that the enzymatic reaction follows the typical Michaelis-Menten mechanism. The RADA16H, RADA16H_2_, and RADA16H_3_ catalyzed the hydrolysis of pNPA with a constant Km on the order of 3 × 10^-3^ M, with k_cat_ values of 2.43 × 10^-3^ s^−1^ -4.87 × 10^-3^ s^−1^ (Supplementary Table S2). The catalytical efficiencies (k_cat_/K_m_) of RADA16H, RADA16H_2_, and RADA16H_3_ were on the same order of most of the previously reported artificial peptide-based enzymes for pNPA hydrolysis (Singh et al., 2015; Zhang et al., 2017; Huang et al., 2020; Liu et al., 2020; Hamley 2021; Singh et al., 2021). However, it is 40-240 times slower than the histidine-containing amyloid-forming peptides, such as IHIHIQI, IHIHIQI and IHIHIYI, which include a Zn^2+^ in their catalytic center (Rufo et al., 2014; Al-Garawi et al., 2017; Song et al., 2018).

The hydrolytic activities of these catalytic nanofibers was likely due to the presence of high densities of reactive histidine residues on the surface of the β-sheet nanostructures with a significant internal order that can reduce the entropic penalty of substrate binding (Zhang et al., 2017; Hamley 2021; Wang et al., 2021). As expected, the catalytical efficiency of RADA16H_2_ was significantly higher than that of RADAH, suggests that more histidine residues exposed for the hydrolysis of pNPA at the edge of the peptide nanofibers. However, as compared with RADA16H_2_, only slightly increase in catalytic efficiency was seen for RADA16H_3_ and indicates that the peripheral histidine of RADA16H_3_ may be located away from the catalytic center and thus has little contribution to the hydrolytic activity. These results demonstrate that the spatial distance between the histidines and substrate binding site may play a key role in the catalytical activities of the histidine-bearing peptides. Concurrently, RADA16 did not show any detectable activity (Supplementary Table S2). The control groups 4-methylimidazole and RGDA16H_2_ had no such kinetic pattern. Their hydrolytic rates were linearly related to the concentration of the substrate (Supplementary Figure S3). Interestingly, the catalytic rate of 4-methylimidazole was higher than that of RGDA16H_2_, although the two histidines were incorporated into the RGDA16H_2_ molecules. The rate decrease could be explained by the poor affinity of substrate to the polydisperse spherical aggregates, which are expected have much less internal order.

RADA16H_2_ nanofibers were quite stable in a wide range of pH values (Supplementary Figures S4A–C). The pH-dependence of RADA16H_2_ catalyzed pNPA hydrolysis showed that the catalytic rate of RADA16H_2_ was positively correlated to pH and exhibited a strong increase between pH 9.0-10.1 (Supplementary Figure S5). AFM morphological studies revealed that the statistical nanofibers height increased significantly from 4.01 ± 0.74 to 8.76 ± 2.66 nm, suggesting that RADA16H_2_ nanofibers might further assemble into fibre bundles which consist of stacked peptide bilayers upon increasing pH from 9.0 to 10.1 (Supplementary Figure S4D) (Sawaya et al., 2007; Bagrov et al., 2016). This observation again indicates that the assembled structure of the peptide contributed significantly to the catalytic activity.

### Substrate specificity of the peptide nanofibers

Carboxylesterase (EC 3.1.1.1) and lipases (EC 3.1.1.3) are well-known lipolytic enzymes catalyzing the cleavage and formation of ester bonds. Even though they share similar architectures and catalytic mechanisms, their substrate specificities for the acyl moiety differ. Esterases typically show a preference for short-chain acyl esters with acyl chain lengths less than 10 carbon atoms (Chang et al., 2021). Some esterases have a narrower substrate specificity that is highly specific for fatty acid esters containing 2 to 4 carbon atoms (Alvarez-Macarie et al., 1999; Levisson et al., 2009; Levisson et al., 2012; Mohamed et al., 2013; Shao et al., 2013; Zhu et al., 2013; Wang M. et al., 2016). However, lipases catalyze the hydrolysis of long-chain acyl esters.

**FIGURE 4:**
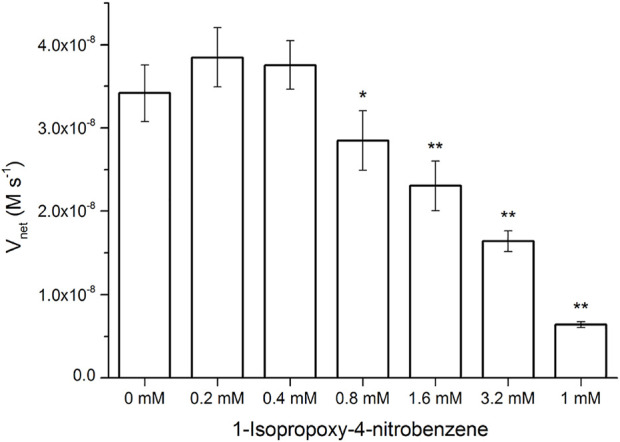
Hydrolysis rate of 0.2 mM of p-nitrophenyl esters with alkyl groups of various length (C2 to C14) by 0.1 mM of RADA16H_2_ at 25°C. *p < 0.001 vs. pNPA and ^#^ *p* < 0.001 vs. pNPB, respectively.

To further test the substrate specificity of the designed peptides, the catalytic capacity of RADA16H_2_ as a function of acyl chain length was studied from C2 to C14 using various p-nitrophenyl esters. The RADA16H_2_ showed preferential hydrolytic activity towards short chain acids esters containing 2 to 4 carbon atoms with a maximum hydrolysis rate for pNPA (pNPC2) (Figure 4; Supplementary Figure S6). The hydrolysis rate was markedly decreased (less than 20% of the maximum) in esters with an acyl chain length longer than C4. These results indicate that these custom peptides have an esterase-like specificity rather than lipase-like pattern.

### The inhibitory effects of the pNPA resembling compounds

**FIGURE 5:**
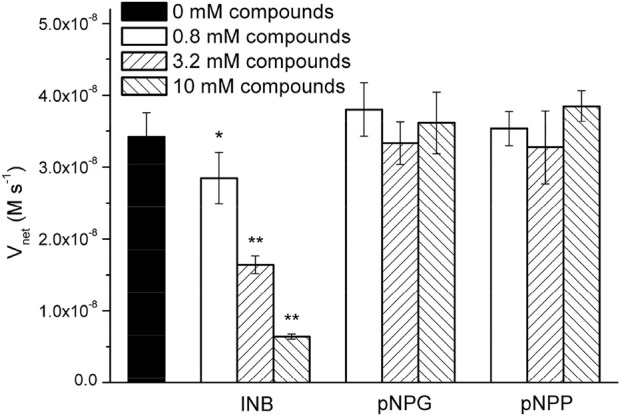
Hydrolysis rate of 0.4 mM of pNPA in the presence of various concentrations of INB, pNPG, and pNPP by 0.1 mM of RADA16H_2_ at 25°C. *p < 0.05, **p < 0.001 vs. the hydrolysis rate of pNPA without any pNPA-mimicking compound.

Surprisingly, the K_m_ parameters for p-nitrophenyl esters (Supplementary Table S2) were very similar to natural enzymes such as esterases from bacteria and archaea (Levisson et al., 2009; Shao et al., 2013; Zhu et al., 2013; Huang P.-S. et al., 2016), although their experimental conditions were different from those used in the present study. These peptide nanofibers exhibit relatively good affinity for the substrate. Natural enzymes are frequently inhibited by compounds resembling the substrate molecule. Thus, we assayed the inhibitory effects of INB, p-nitrophenyl glycerol (pNPG), and pNP phosphate (pNPP) (Supplementary Scheme S1), which are structural similar to pNPA. The inhibitory assay showed that 0.8 mM of the hydrophobic INB efficiently inhibited the hydrolysis of pNPA by RADA16H_2_ (Supplementary Figure S7). No significant inhibition was detected with pNPG and pNPP at concentrations up to 10 mM, suggesting that the peptide nanofibers preferentially bind to hydrophobic nitrophenyl molecules rather than hydrophilic or charged ones (Figure 5). These results confirmed the presence of the hydrophobic binding sites. Indeed, the formation of hydrophobic surfaces on the β-sheet may play a crucial role in the binding of the hydrophobic substrate.

**FIGURE 6:**
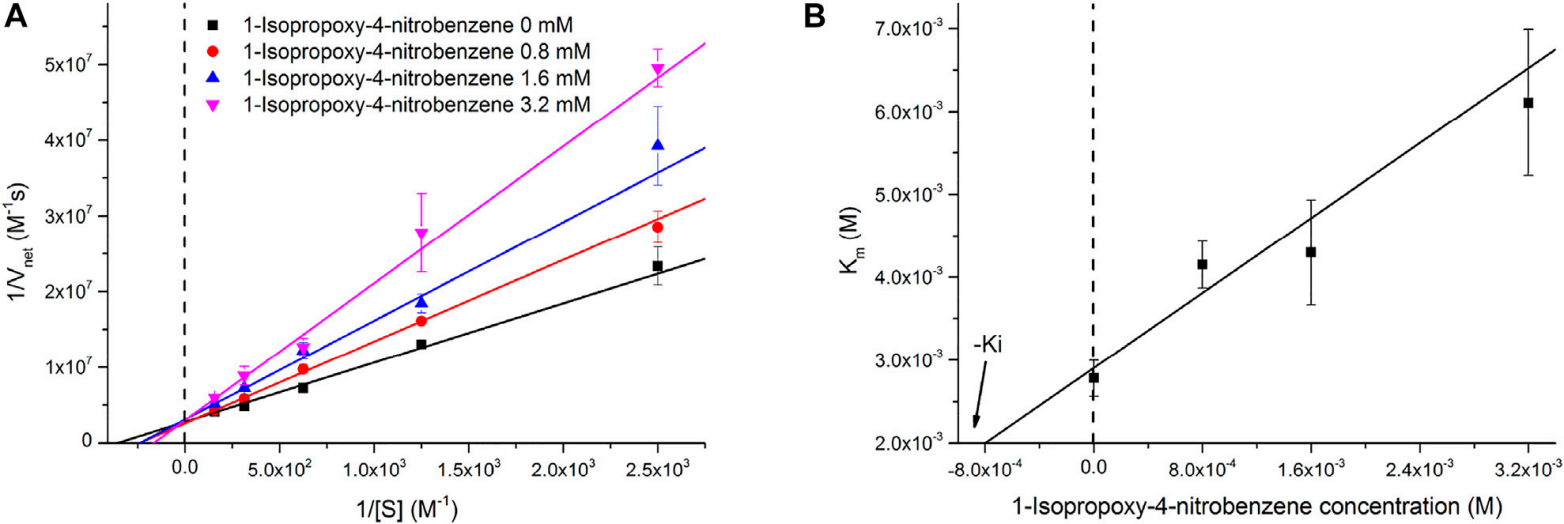
**(A)** Lineweaver-Burke plots of the hydrolysis of pNPA catalyzed by 0.1 mM of RADA16H_2_ at 25°C in the presence of INB as inhibitor. These plots are straight lines with similar slopes suggesting a competitive inhibition of RADA16H_2_ with INB. **(B)** Dixon plot for the inhibitory effect of INB on the RADA16H_2_ catalysis reaction.

The inhibitory effect of INB on the RADA16H_2_ nanofibers and the catalytic reaction of pNPA were evaluated further. The hydrolysis of pNPA catalyzed by RADA16H_2_ was inhibited by INB in a dose-dependent manner when the concentration of INB exceeded 0.4 mM, and double-reciprocal plots are shown in Figure 6A. The plots were obtained by varying the concentration of the inhibitor from 0 to 3.2 mM. The plots of a group of straight lines with similar intercepts show hallmark features of a competitive inhibition (Arsalan and Younus, 2018), suggesting that INB competes with pNPA for binding to the nanofibers (Sharma et al., 2018). Dixon analysis was used with varying concentrations of INB to yield an inhibition constant (K_i_) of 2.57 mM (Figure 6B).

There are two overarching features of the results to emphasize. First, it is confusing how the simple structure of the β-sheet nanofibers can display such a high esterase-like specificity. Second, the mechanism of the competitive inhibition is unclear. We believe that these two features could be interpreted by the non-specific hydrophobic interaction between the edge of the bilayer fibrils and the hydrophobic substrate with various alkyl chains. The spatial distance between the histidines and hydrophobic edge may decide the substrate specificity of the artificial esters. Long acyl chains (over 4 carbons atoms) potentially hamper the approach or direction of the substrate to the active site. In addition, INB competes with the binding of pNPA at higher concentrations (>0.4 mM) because of non-specific binding. Since INB binds reversibly, the inhibition can be overcome by increasing the pNPA concentration. This results in an apparent increase in K_m_ without change in k_cat_ (Supplementary Table S3).

## Conclusion

In conclusion, short peptides were designed from amphiphilic nanofibers for efficient catalysis of p-nitrophenyl ester hydrolysis with remarkable substrate selectivity. The histidine residues extend from the nanofibers with significant internal order and serve as active sites for ester hydrolysis. The inhibition experiments suggest that the hydrophobic edges of the self-assembled fibrillar structure plays a crucial role in the binding of hydrophobic substrate. We note that these peptides exhibit substrate specificity for short-chain acids esters and esterase-like catalytic behavior in addition to the lipase-like activity. Finally, a pNPA-resembling hydrophobic compound (INB) was found to be a competitive inhibitor for pNPA hydrolysis. This approach is a promising method for developing enzyme mimics with substrate specificity using self-assembling peptides as the supramolecular framework.

## Data Availability

The raw data supporting the conclusion of this article will be made available by the authors, without undue reservation.
