# Supplementary material for: An artificial self-assembling peptide with carboxylesterase activity and substrate specificity restricted to short-chain acid p-nitrophenyl esters
Source: Front Chem. 2022 Sep 19;10:996641. doi: 10.3389/fchem.2022.996641 (PMC9527324; doi:10.3389/fchem.2022.996641)
Supplement: Supplementary file 1 [file DataSheet1.docx]

Supplementary Material





**Scheme S1.** Chemical structures of compounds resembling pNPA used in the inhibition experiment.

**

**

**Figure S1.** The storage modulus (a) and loss modulus (b) of the hydrogel formed by 5 mM of RADA16H, RADA16H_2_, RADA16H_3_ and RGDA16H_2_ with 50 mM PBS buffer at 25 °C.


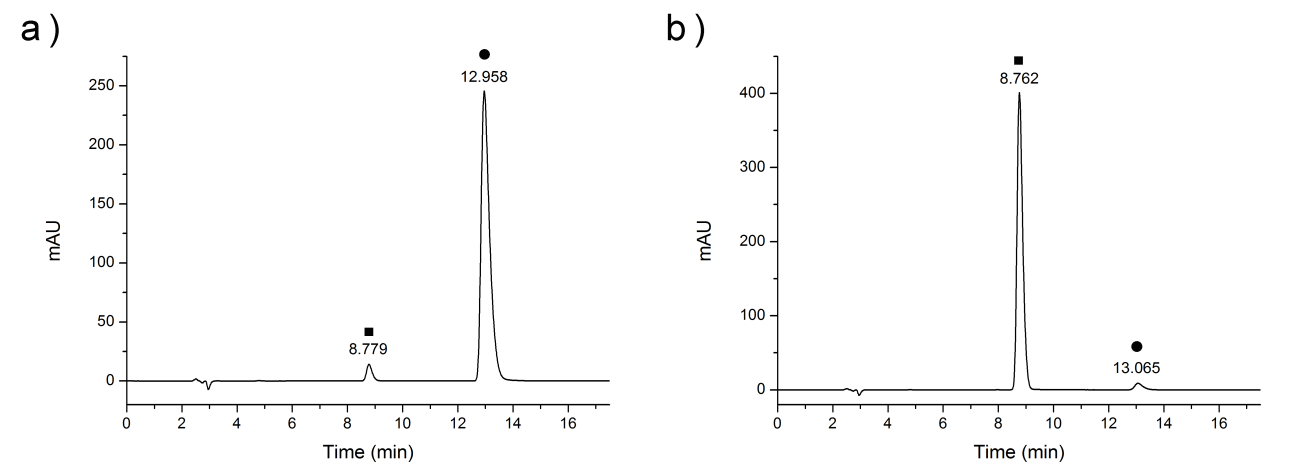


**Figure S2.** HPLC chromatogram of pNPA (●) and product p-nitrophenol (■) in the reagents at (a): 0 h and (b): 6 h.





**Figure S3.** Hydrolysis rate of pNPA by 0.1 m M of RGDA16H_2_ and 4-methylimidazole at 25 °C.


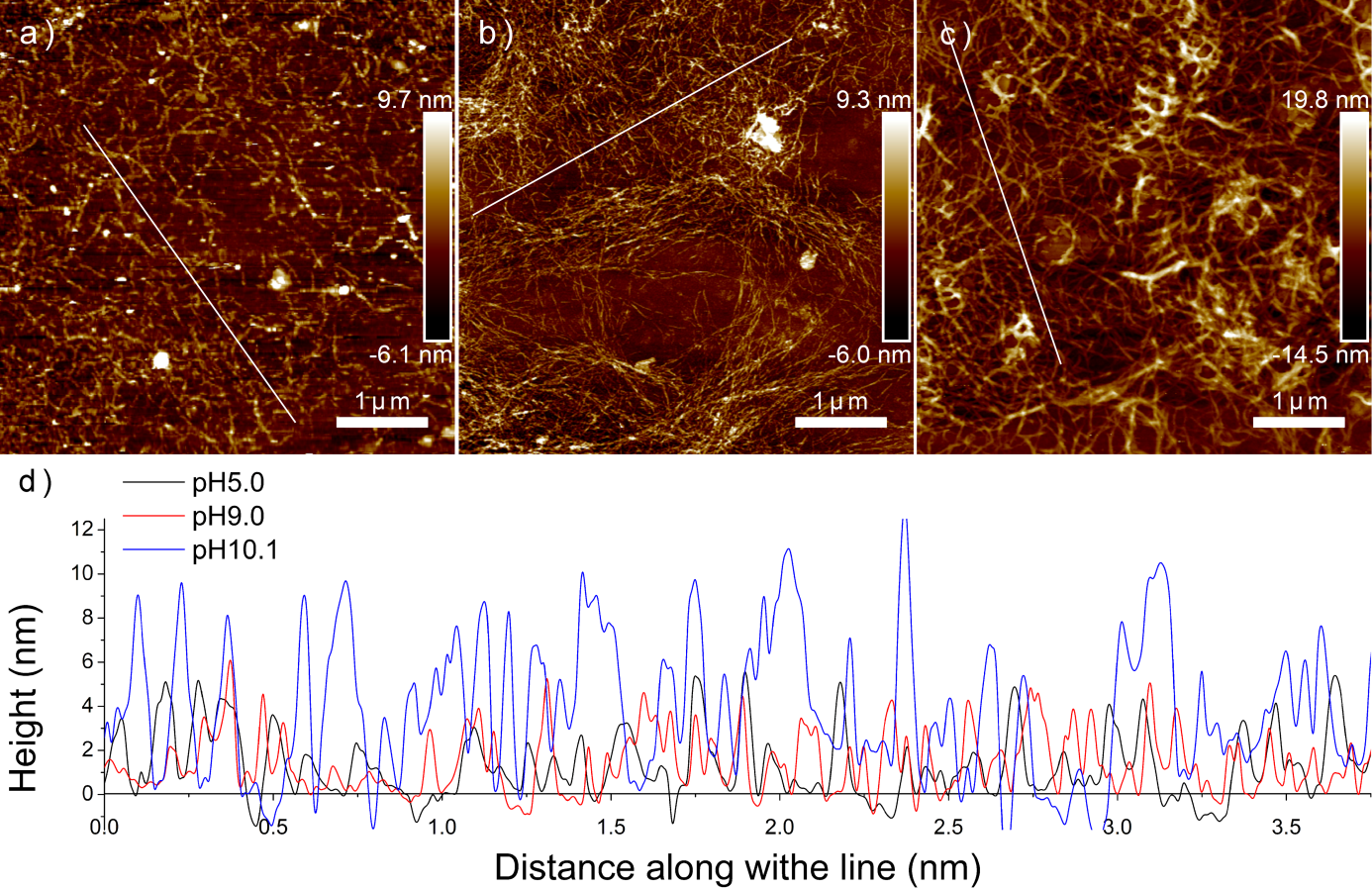


**Figure S4.** Tapping mode AFM images of 1 mg/mL RADA16H_2_ nanofibers formed at (a) pH5.0, (b) pH9.0 and (c) pH10.1. (d) White lines point out the measured cross-section height of the nanofiber network.





**Figure S5.** (a) pH dependency of the k_cat_ for pNPA hydrolysis by 0.1 mM of RADA16H_2_ at 25 °C. *The hydrolysis rate at pH 5.0 was too slow to obtain the accurate parameters. (b and c) The background hydrolysis profiles of pNPA at different pH values.





Figure S6. Plots of absorbance vs time for hydrolysis of 0.2 mM of p-nitrophenyl esters with alkyl groups of various length (C2 to C14) by 0.1 mM of RADA16H_2_ at 25°C.





**Figure S7.** Hydrolysis rates of pNPA by RADA16H_2_ in presence of various concentrations of INB at 25 °C. **P*<0.05 and ***P*<0.005 compared with hydrolysis rate of pNPA without INB.


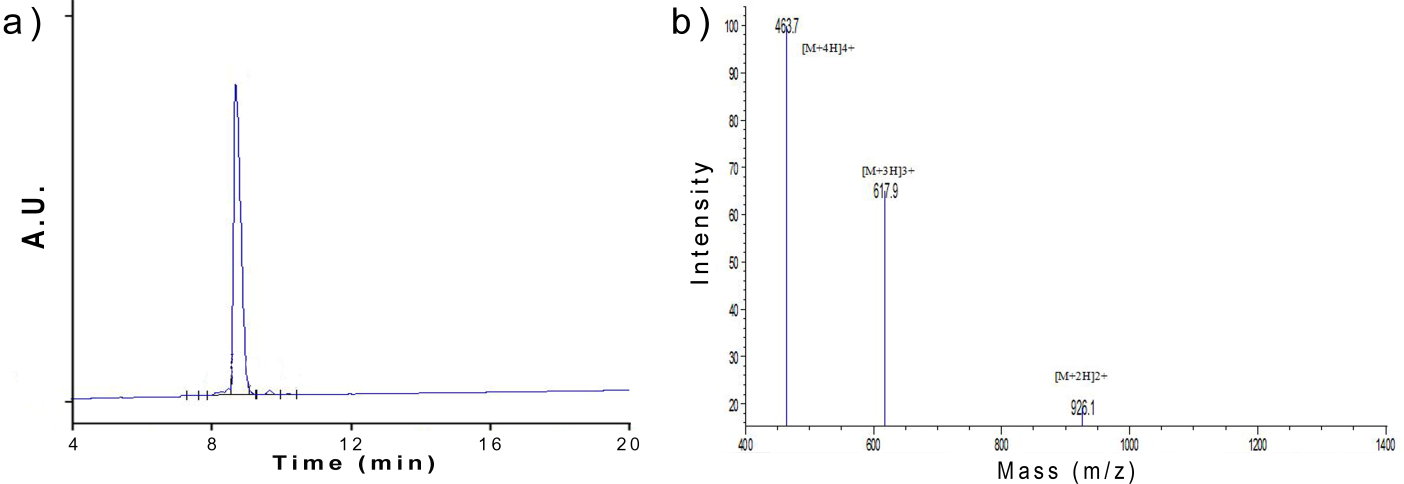


**Figure S8.** HPLC chromatogram and MALDI-TOF MS of peptide RADA16H.


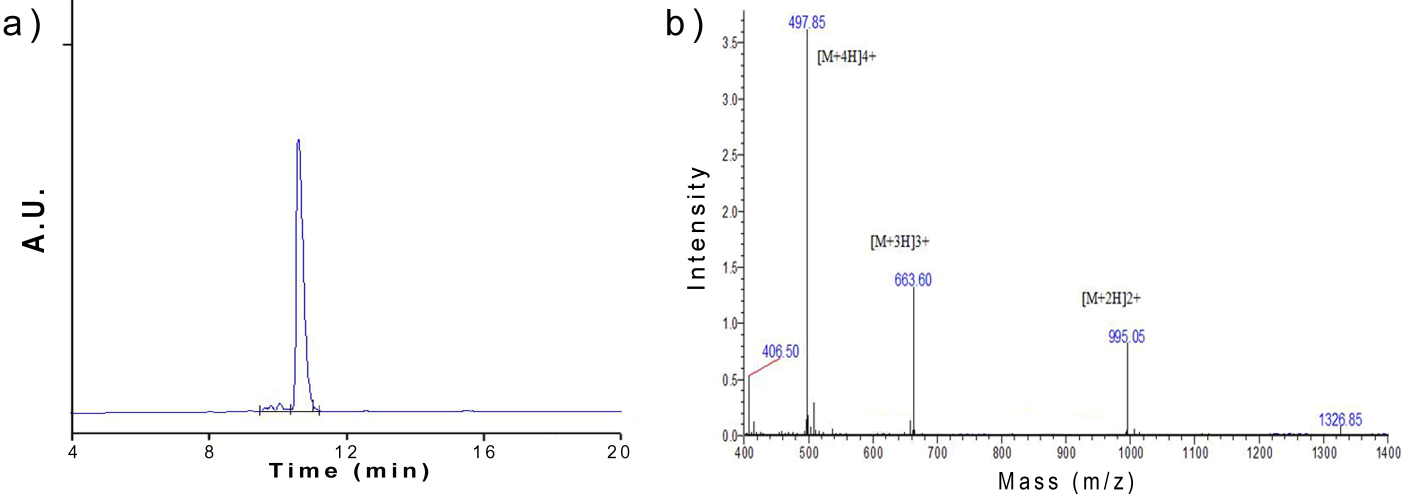


**Figure S9.** HPLC chromatogram and MALDI-TOF MS of peptide RADA16H_2_.


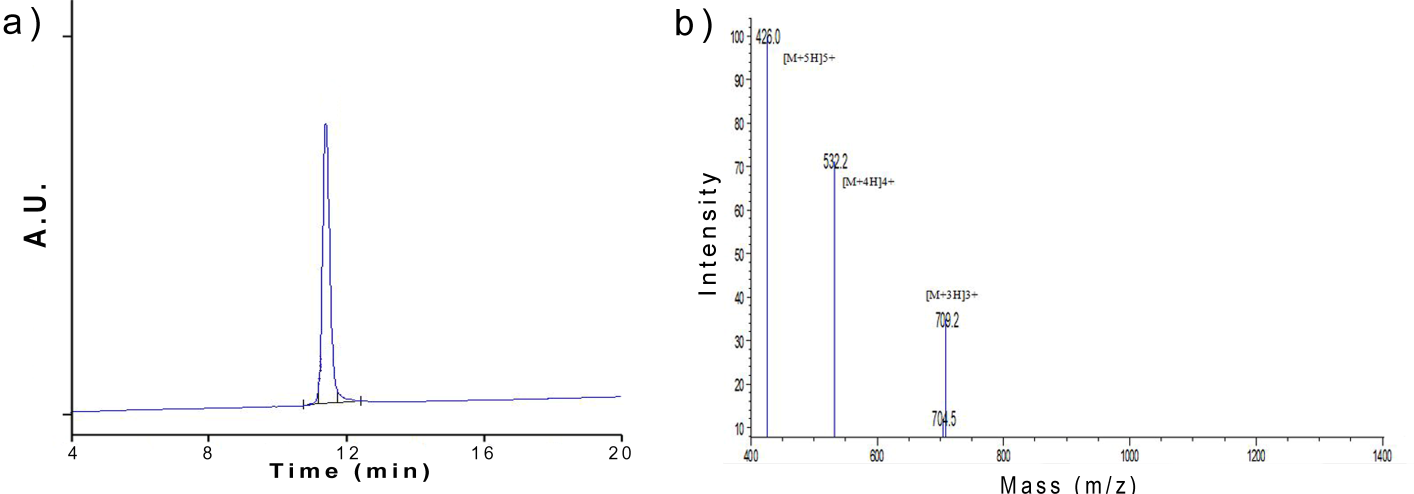


**Figure S10.** HPLC chromatogram and MALDI-TOF MS of peptide RADA16H_3_.


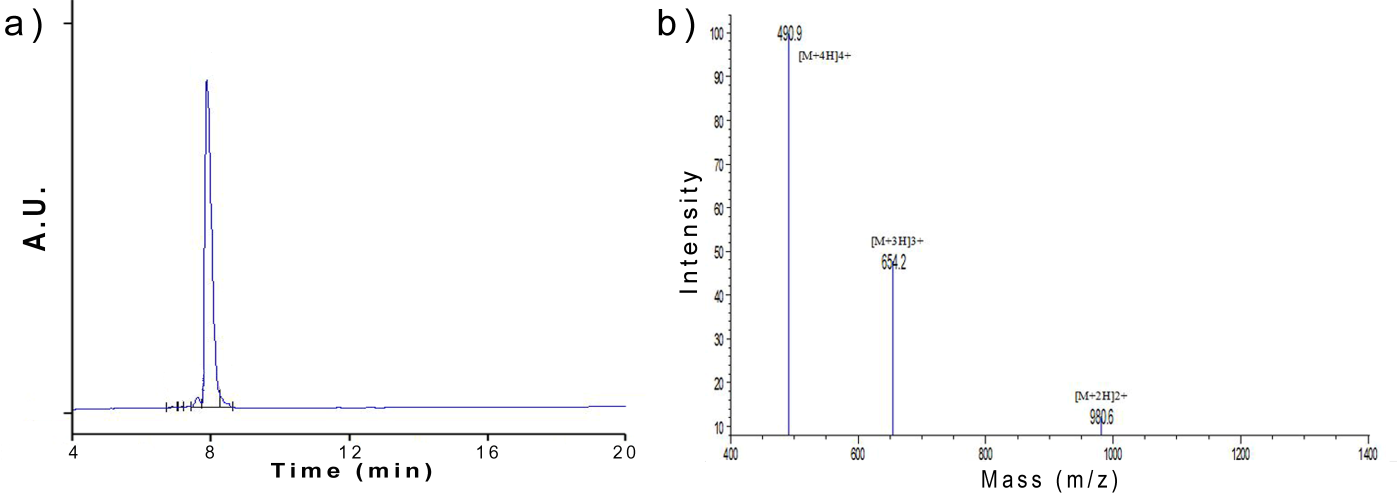


**Figure S11.** HPLC chromatogram and MALDI-TOF MS of peptide RGDA16H_2_.

| **Table S1.** Estimated structure fractions of the peptides in aqueous solution | | | | | | |
| --- | --- | --- | --- | --- | --- | --- |
| Peptide | Secondary structure fractions (%) | | | | | |
|  | H(r) | H(d) | S(r) | S (d) | Turn | Unrd |
| RADA16H | 0.137 | 0.118 | 0.382 | 0.219 | 0.394 | 0.355 |
| RADA16H_2_ | 0.090 | 0.090 | 0.269 | 0.157 | 0.280 | 0.352 |
| RADA16H_3_ | 0.080 | 0.096 | 0.217 | 0.130 | 0.248 | 0.494 |
| RGDA16H_2_ | 0.003 | 0.028 | 0.058 | 0.035 | 0.069 | 0.234 |
| H (r), regular [α](https://mc.manuscriptcentral.com/jocl?PARAMS=xik_CgdjHbYgpj28Ex1vkzCND3XBcYcq3opg7X9hQNQh9XBp8CVme6fCyQMZcXUy5cCW7zgTyBzca4JVfrArfGJKc1Tcc7UKBMZtTbHj353bXurqXHaPAijBNZmendgNEqZXcRNG5gcVNVs2kegGToV84ptZMWL6PbUHYUgY5LBip4mkVdaBpWhmSgQfiEDHe7W3VfRpF)-helix; H (d), distorted [α](https://mc.manuscriptcentral.com/jocl?PARAMS=xik_CgdjHbYgpj28Ex1vkzCND3XBcYcq3opg7X9hQNQh9XBp8CVme6fCyQMZcXUy5cCW7zgTyBzca4JVfrArfGJKc1Tcc7UKBMZtTbHj353bXurqXHaPAijBNZmendgNEqZXcRNG5gcVNVs2kegGToV84ptZMWL6PbUHYUgY5LBip4mkVdaBpWhmSgQfiEDHe7W3VfRpF)-helix; S (r), regular β-strand; S (d), distorted β-strand (a partial but far-from-complete distortion of the regular β-strand, due to lack of some hydrogen bonds); Turn, β-turn structure; Unrd, unordered structure. | | | | | | |

| **Table S2.** Kinetics constants for catalyzed hydrolysis of pNPA | | | | |
| --- | --- | --- | --- | --- |
|  | k_cat_/s^-1^ | K_m_/M | k_cat_/ K_m_/M^-1^s^-1^ | K_2_/M^-1^s^-1^ |
| RADA16H | 2.43±0.18×10^-3^ | 3.41±0.45×10^-3^ | 0.71 | - |
| RADA16H_2_ | 3.55±0.19×10^-3^ | 2.78±0.22×10^-3^ | 1.28 | - |
| RADA16H_3_ | 4.87±0.21×10^-3^ | 3.35±0.22×10^-3^ | 1.45 | - |
| RGDA16H_2_ | - | - | - | 0.09 |
| RADA16 | - | - | - | - |
| 4-MeIm | - | - | - | 0.18 |
| The kinetics constants given are derived from the Michaelis–Menten equation.  All the experiments were repeated three times. Note that RADA16 did not show any detectable activity. | | | | |

…

| **Table S3.** Kinetics constants for RADA16H_2_ catalyzed hydrolysis of pNPA in the presence of INB as inhibitor. | | | |
| --- | --- | --- | --- |
| INB concentration/mM | k_cat_/s^-1^ | K_m_/M | k_cat_/ K_m_/M^-1^s^-1^ |
| 0 | 3.55±0.19×10^-3^ | 2.78±0.22×10^-3^ | 1.28 |
| 0.8 | 3.84±0.22×10^-3^ | 4.15±0.28×10^-3^ | 0.93 |
| 1.6 | 3.26±0.32×10^-3^ | 4.30±0.63×10^-3^ | 0.76 |
| 3.2 | 3.34±0.38×10^-3^ | 6.11±0.88×10^-3^ | 0.55 |
| The kinetics constants given are derived from the Michaelis–Menten equation. All the experiments were repeated three times. | | | |

| **Table S4.** Kinetics constants as a function of pH for the RADA16H_2_ catalyzed hydrolysis of pNPA. | | | | | |
| --- | --- | --- | --- | --- | --- |
| pH | Buffer | k_cat_/s^-1^ | pH | Buffer | k_cat_/s^-1^ |
| 5.0^[a]^ | Acetate | - | 8.2 | Borate | 4.77±1.03×10^-3^ |
| 6.5 | Phosphate | 1.44±0.25×10^-3^ | 9.0 | Borate | 5.52±1.55×10^-3^ |
| 7.4 | Phosphate | 3.55±0.19×10^-3^ | 10.1 | Borate | 12.42±1.97×10-^3^ |
| The kinetics constants given are derived from the Michaelis–Menten equation. The buffer concentrations were 50 mM. ^[a]^ The hydrolysis rate was too slow to obtain the accurate parameters. | | | | | |
